# Elastic fixation of mallet finger fractures using two K-wires: A case report of a new fixation technique

**DOI:** 10.1097/MD.0000000000015481

**Published:** 2019-05-17

**Authors:** Qiang Chen, Yan Suo, Danhong Pan, QingPing Xie

**Affiliations:** Department of Hand Surgery, Zhejiang Provincial People's Hospital, People's Hospital of Hangzhou Medical College, Hangzhou, Zhejiang, P.R. China.

**Keywords:** bony fragment, elastic fixation, K-wire, mallet finger, surgical treatment

## Abstract

**Rationale:**

Mallet finger fracture is a common sports-related injury that may lead to the tearing of extensor tendon and protrusion of a bony fragment located at the base of the distal phalanx. We affirmed that the elastic fixation of with two K-wires technique is a good method to deal with Mallet Finger fractures that fractures could gain effective fixation than the conventional treatment method and avoid surgical incision complication

**Patient concerns:**

We reported a 33-year-old female patient came to our hospital complaining of mild pain, swelling and her right little finger was deformed because of sport's injury.

**Diagnosis:**

Acute mallet finger fracture type IV B according to Doyle classification of mallet injuries.

**Interventions:**

We performed an emergency operation for the elastic fixation of the mallet finger fractures with two K-wires.

**Outcomes:**

After the surgery, the patient showed functional recovery. No evidence of recurrence was noted 6 months after the operation, and the patient showed no symptoms of sports-related injuries.

**Lessons:**

We discuss the clinical diagnosis, treatment, and follow-up of the patient and suggest that elastic fixation with two K-wires is a good method to treat mallet finger fractures.

## 1 Introduction

In clinical practice, mallet finger fractures due to sports-related injuries are commonly encountered. Mallet finger fractures may lead to the tearing of the extensor tendon attached to a bony fragment located at the base of the distal phalanx.^[1]^ If the patient does not receive effective treatment, the distal phalanx may gradually assume a fixed flexed position. Sometimes, the proximal phalangeal joints may gradually hyperextend. Various treatment strategies have been studied, but the optimal treatment choice is not clear.^[2–4]^

Conservative treatment for mallet finger fracture includes continuous aluminum splinting and application of splints, plaster casts, and orthosis brace for at least 6 weeks.^[5]^

However, nonsurgical treatment is not suitable for patients with an intra-articular avulsion fracture of more than one-fourth of the base of the distal phalanx. Surgery is indicated in such situations.^[6]^ Various surgical techniques have been described, including open reduction, K-wire fixation, pin fixation alone, tension band wire fixation, and pull-out steel wire fixation pin migration, loss of reduction, and avascular necrosis of the fragment are major complications, according to the type of surgery.^[7,8]^ Some experts admired the value of minimally invasive closed reduction of mallet fractures by K-wire.^[9]^ Here we report a new fixation technique for mallet fractures using two K-wires to accomplish elastic fixation of mallet finger fractures. To date no articles reporting this kind of treatment have referred to the same patient.

## 2 Case presentation

**Figure 1:**
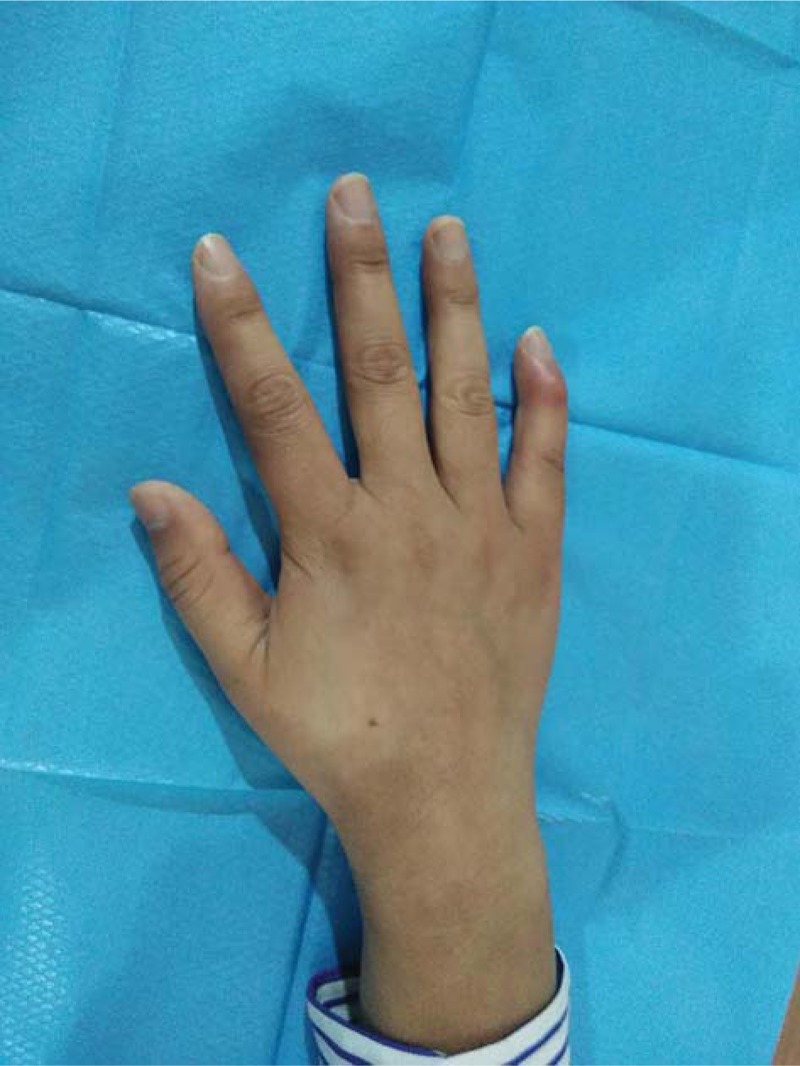
Photo of hand pre-operation.

**Figure 2:**
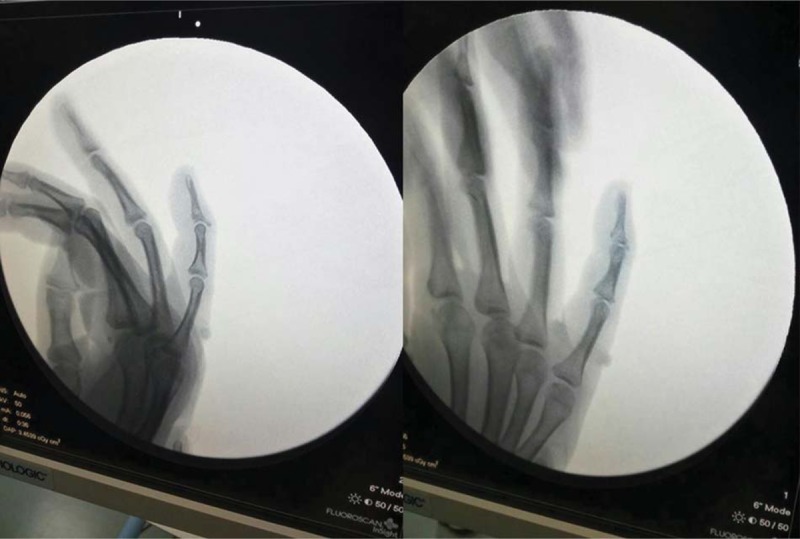
X-ray images were taken before the operation revealed that there is a avulsion fracture of more than one-fourth of the base located on the distal intra-articular.

**Figure 3:**
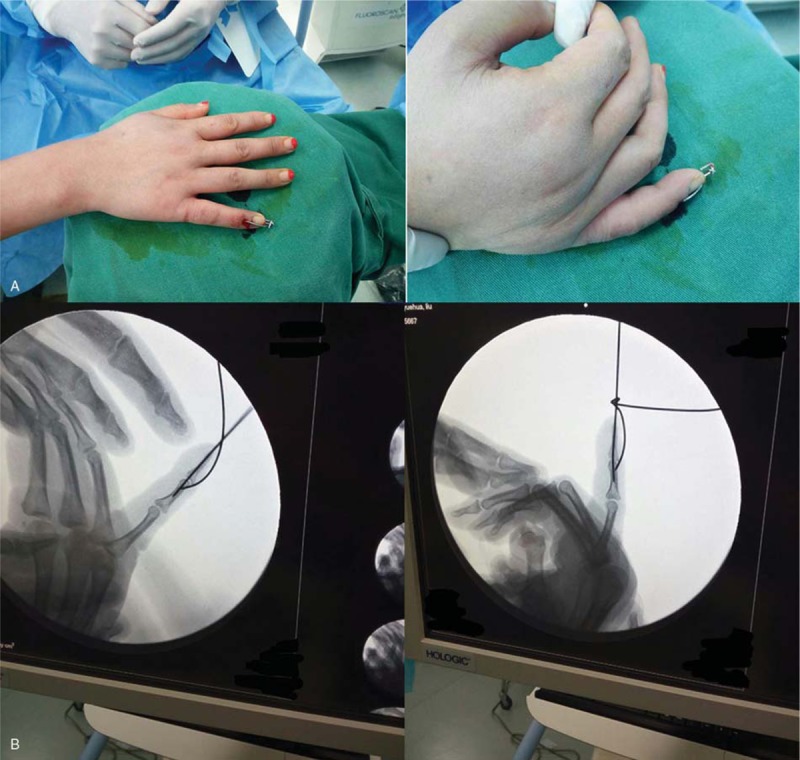
A: Photographs of the finger post-operation. B: X-ray images of the finger post-operation.

A 33-year-old female patient visited the Hand Department with complaints of mild pain and swelling. The little finger of her right hand was deformed because of an injury in a amateur basketball match 1 day prior. She requested for an emergency operation immediately. Upon inspection, she could not straighten the distal phalanx of her right little finger completely; the finger had assumed a fixed flexed position. The local general surgeon had used a splint to keep the finger extended completely. Unfortunately, the skin of wound bruised (Fig. 1), and the incision may have turned necrotic after operation; therefore, open reduction was not a viable option. The patient was referred to the Hand Department. Her laboratory test findings were normal; however, further examination by X-ray imaging revealed that an intra-articular avulsion fracture of more than one-fourth of the base of the distal phalanx (Fig. 2) that appeared to be type IV B injury, according to Doyle's classification.^[7]^ Considering the patient's condition, we decided to fix the bony fragment by K-wire fixation, which is a minimally invasive procedure with no skin incision. The surgical procedure was designed to first insert a 0.8 mm diameter K-wire from the dorsal side of the bony fragment to the middle phalanx, as close to the bony fragment as possible. The second 0.8 mm-diameter K-wire was inserted from the tip of the distal phalanx to the middle phalanx so that the finger is distally slightly hyperextended. We bended the first K-wire to cross the second K-wire to first reset the avulsion fracture. With the increase in angle, the avulsion fracture was able to undergo anatomy reduction gradually. When confirming fracture reset under the screen of the C-arm X-ray equipment we could tie up the two K-wires around each other using a steel wire at the end (Fig. 3). The ends of the two K-wires protrude out of the skin (2–3 mm). A small splint over the metacarpophalangeal joint was used for 2 weeks followed by a finger plaster cast applied directly over the DIP joint but allowed active motion of the proximal interphalangeal and metacarpophalangeal joints. The suture line was removed 2 weeks after surgery. When the cortices of the fragment appeared to be joined on the radiographs, we confirmed that union of the fracture had occurred. Then both K-wires were removed under a digital nerve block 6 weeks after surgery.

**Figure 4:**
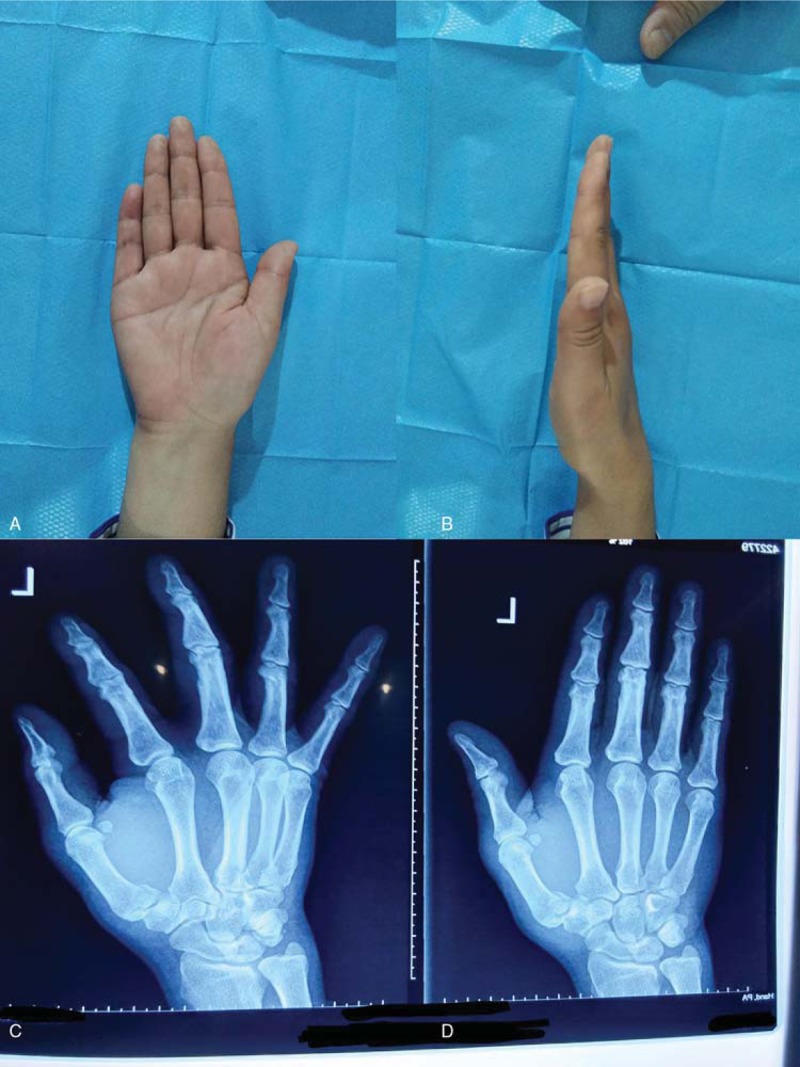
follow-up (A and B) postoperative follow-up 3 months after surgery showed excellent movements (C and D) X-ray radiography shows that the fracture healed well.

The swelling in the surgical area was gradually reduced after the operation and returned to normal 3 days later. Six weeks after the surgery, X-ray scans both revealed no evidence of displacement. The patient also reported no finger pain or swelling symptoms. Six weeks after the surgery we pull out two K-wires and 3 months later operation follow up showed the finger returned to normal movement. Nevertheless, we continued to observe and follow up with the patient (Fig. 4).

This study protocols were approved by the medical ethics committee of the Zhejiang Provincial People's Hospital. Written informed consent for publication of clinical details and images was obtained from the patient. A copy of the consent form is available for review upon request.

## 3 Discussion

In clinical practice, mallet finger fracture is commonly encountered due to work accidents and sports injuries, depending on the increasing frequency of sports activities and home accidents.^[8]^ The mechanism of injury for mallet fractures is usually axial loading of the DIP joint. Displaced mallet fractures that are not treated well may result in extension lag and swan neck deformities in some cases.^[10]^ The study by Jillian S. Gruber study showed that some of the displaced bony mallets have decreased mechanical advantage and more marked residual droop and extensor lag.^[11]^ Mallet fractures, especially those with associated subluxation of the distal phalanx, are type IV mallet finger injuries, as defined by Doyle's classification, that most surgeons recommend be treated by reduction operation.^[12]^ Surgical treatments include Kirschner (K)-wire pinning of the fracture fragments or DIP joints,^[13]^ suturing,^[14]^ tension band wiring,^[15]^ extension block pinning,^[16–18]^ and plating.^[19]^

Each surgical treatment is associated with risks of infection, necrosis of skin and cutaneous, and nonunion or malunion of the fractured fragments. Thus, various studies certified that there is no gold standard to treat mallet fractures.^[20]^

Wenlong Zhang et al had reported their new fixation technique for mallet fractures of pressing fixation with the end of a K-wire.^[21]^ Dong Hee Kim also have the similar “Fish Hook ” technique for mallet finger.^[22]^ On the basis of their surgical methods, we improved the procedure on some points. The goal of this report is to present a simple method of two K-wires fixation method and show our results with this procedure.

Closed treatment has provided good results in uncomplicated cases of mallet finger; however, surgical fixation is recommended when there is involvement of more than one-fourth of the base of the distal phalanx. Various techniques have been described for this purpose.

The purpose of this study is to present a case report of patient treated with a new K-wires elastic fixation technique for the management of mallet fractures.

The first advantage of the technique is that we had no incision and we only insert K-wire to force fracture fragments reset so as to avoiding skin necrosis. It is suitable for those patients whose skin of wound was bruised and markedly swollen. Secondly, according to Zhang et al, the K-wire hook end should press the center of the fragment. If the hook pressed the far-end or near-end of the fragment, the fragment will shift or even overturn under the tension.^[21]^ When confirming fracture reset under the screen of the C-arm X-ray, we could tie up the two K-wires around each other using a steel wire at the end. Using our method, the fragment will not shift easily and will hold the fragment anatomically in position to ensure a good fracture union. The patient gained functional recovery postoperatively after 3 months.

The study has certain limitations, like the absence of a comparison group. We plan to conduct prospective randomized blinded studies in the future to evaluate the efficacy of our technique.

## Acknowledgments

The author would like to express deep gratitude to my colleagues, especially Dr Qing-PingXie, the leader of my department, as well as Dr Yan Suo and Dr Qi Liu, who provided me with great assistance in every stage of writing this paper. I would also like to thank the Zhejiang Medicine and Hygiene Research Program for sponsoring our research (grant number 2019KY025). Zhejiang Traditional Chinese Medicine.

Research Program (grant number2019ZA008).

## Author contributions

**Writing – original draft:** Qiang Chen, Yan Suo, DanHong Pan.

**Writing – review & editing:** QingPing Xie.
